# Partial orientation retrieval of proteins from X-ray free-electron-laser induced explosions for single particle imaging

**DOI:** 10.1038/s41598-025-23827-w

**Published:** 2025-10-23

**Authors:** Tomas André, Alfredo Bellisario, Emilano De Santis, Nicuşor Tîmneanu, Carl Caleman

**Affiliations:** 1 Department of Physics and Astronomy, Uppsala University, Box 516, SE-751 20 Uppsala, Sweden; 2 University of Rome Tor Vergata & INFN, via della Ricerca Scientifica 1, I-00133 Rome, Italy; 3 Center for Free-Electron Laser Science, Deutsches Elektronen-Synchrotron, Notkestraße 85, DE-22607 Hamburg, Germany

**Keywords:** Optics and photonics, Physics

## Abstract

Single Particle Imaging techniques at X-ray lasers have made significant strides, yet the challenge of determining the orientation of freely rotating molecules during delivery remains. In this study, we propose a novel approach to partially retrieve the relative orientation of proteins exposed to ultrafast X-ray pulses by analyzing the fragmentation patterns resulting from Coulomb explosions. We simulate these explosions for 85 proteins in the size range 100 – 4000 atoms using a hybrid Monte Carlo/Molecular Dynamics approach and capture the resulting ion ejection patterns on two virtual detectors. We exploit information from the explosion to infer orientations of proteins at the time of X-ray exposure. Our results demonstrate that partial orientation information can be extracted, particularly for larger proteins. We conclude that knowledge on ion data from X-ray laser induced explosions can directly provide the sample’s relative orientation, complementary to traditional orientation-retrieval algorithms based on diffraction patterns.

## Introduction

Proteins are often described as the building blocks of life due to their fundamental roles in virtually all biological processes across living organisms. Understanding these processes at the molecular level requires detailed knowledge of protein structures, as the structure of a protein is inherently linked to its function. Proteins perform a diverse array of functions, including enzymatic catalysis, signal transduction, and molecular transport, all of which are crucial for maintaining cellular functions. Currently, high-resolution protein structure is often determined through X-ray crystallography, which can resolve the structure to Ångström resolution^1^. Crystallography has some drawbacks, particularly because many important proteins, especially membrane proteins, which constitute a large portion of drug targets^2^ and represent around 30% of all proteins in organisms^3^, are very difficult or even impossible to crystallize due to their structure. This limits the types of sample that can be studied. Furthermore, crystal-induced conformational biases reduce the biological relevance of some structures. A solution to this problem is Single Particle Imaging (SPI)^4^ at X-ray Free Electron Lasers (XFEL), which can image samples as individual non-crystalline particles. SPI is now starting to mature, and there have been significant advancements in the past decade both in sample delivery and analysis methods^5,6^, though further improvements are needed to reach its full potential.

**Fig. 1:**
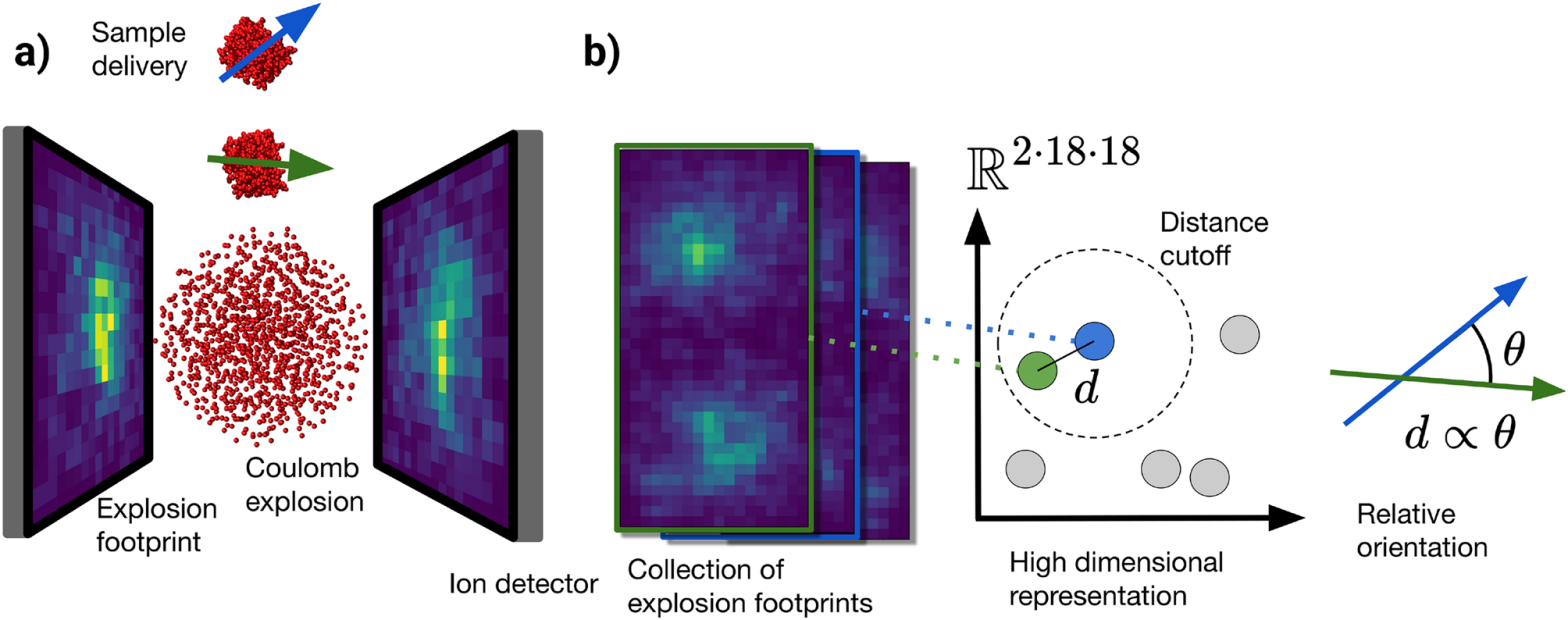
Geometry and setup of our simulated experiment and the pipeline for analysis. (**a**) Two detectors are placed on each side of the incoming particle at the XFEL interaction point, and capture the ejected ions from the exploding sample. Proteins are delivered one at a time with random orientations, represented with green and blue arrows, which physically could be the principal inertia component or dipole moment. (**b**) The explosion footprints are represented as points in a high dimensional space, with a distance $d$. Within a distance cutoff, the distance $d$ is proportional to the relative orientation $\theta$ of the proteins.

One of the challenges that remain to be overcome in SPI is the orientation of the sample^7^. During delivery each molecule can rotate freely in space, and there is currently no way of knowing this orientation. Using laser-induced alignment, there are ways to pre-orient small molecules^8^, and ongoing studies explore pre-orienting larger molecules such as proteins using electric fields during delivery^9–11^. Algorithms such as Expand-Maximize-Compress (EMC)^12^, common arc^13^, and manifold embedding^14^ can reconstruct a sample from diffraction data without any prior knowledge of its orientation; however, they require a large number of high-quality diffraction patterns.

Coulomb explosion imaging is a single-molecule experimental technique for small molecules with around dozens of atoms. In this technique, a sample is heavily ionized, and the fragments are tracked by measuring the momenta of the resulting ions in coincidence^15^. XFELs enable Coulomb explosion imaging because X-rays can be tuned to target specific inner shells while reaching highly charged states via sequential single-photon absorption^16–18^ and have the intensity to strip the electrons from the molecules. XFELs are powerful enough to cause an explosion of a bio-molecule. For small proteins or peptides this explosion has the character of a Coulomb explosion^4^, whereas larger proteins tend to explode in a hydrodynamic manner^19^. For polypeptides, classical Coulomb explosion imaging is not possible since the number of ions makes measuring ions in coincidence unfeasible. However, Östlin *et al.*^20^ simulated X-ray-induced Coulomb explosions on lysozyme to construct time-integrated *explosion footprints* generated by projecting carbon and sulfur ion trajectories onto a virtual spherical detector and concluded these $4\pi$-maps could be used to determine the protein’s orientation during exposure if all ions can be detected. De Santis *et al.*^21^ further studied how the spread of ion trajectories depends on the depth at which the ions are located within the protein at the moment of a hydrodynamic explosion. In our previous works, we proposed the possibility of using explosion patterns to classify proteins solely based on the explosion footprint they create^22,23^. Unlike conventional Coulomb explosion imaging used for very small molecules, the explosion footprints in these studies are simplified: they are constructed only from field free ion trajectories and contain no coincidence, momentum, or mass information. Furthermore, in a recent study, Wollter *et al.*^24^ showed that partial knowledge about protein orientation can improve reconstruction capabilities of EMC as it would reduce the number of diffraction patterns required for successful reconstruction as well as allow the use of noisier patterns.

In the present theoretical work we pose the question: *Can XFEL induced explosion patterns encode orientation information?* Our goal is to describe a method for inferring partial orientation information from ion explosions footprints. This approach could provide valuable knowledge to current orientation retrieval algorithms and provide an alternative solution when the number of patterns is limited or the signal-to-noise ratio in diffraction measurements is low.

**Fig. 2:**
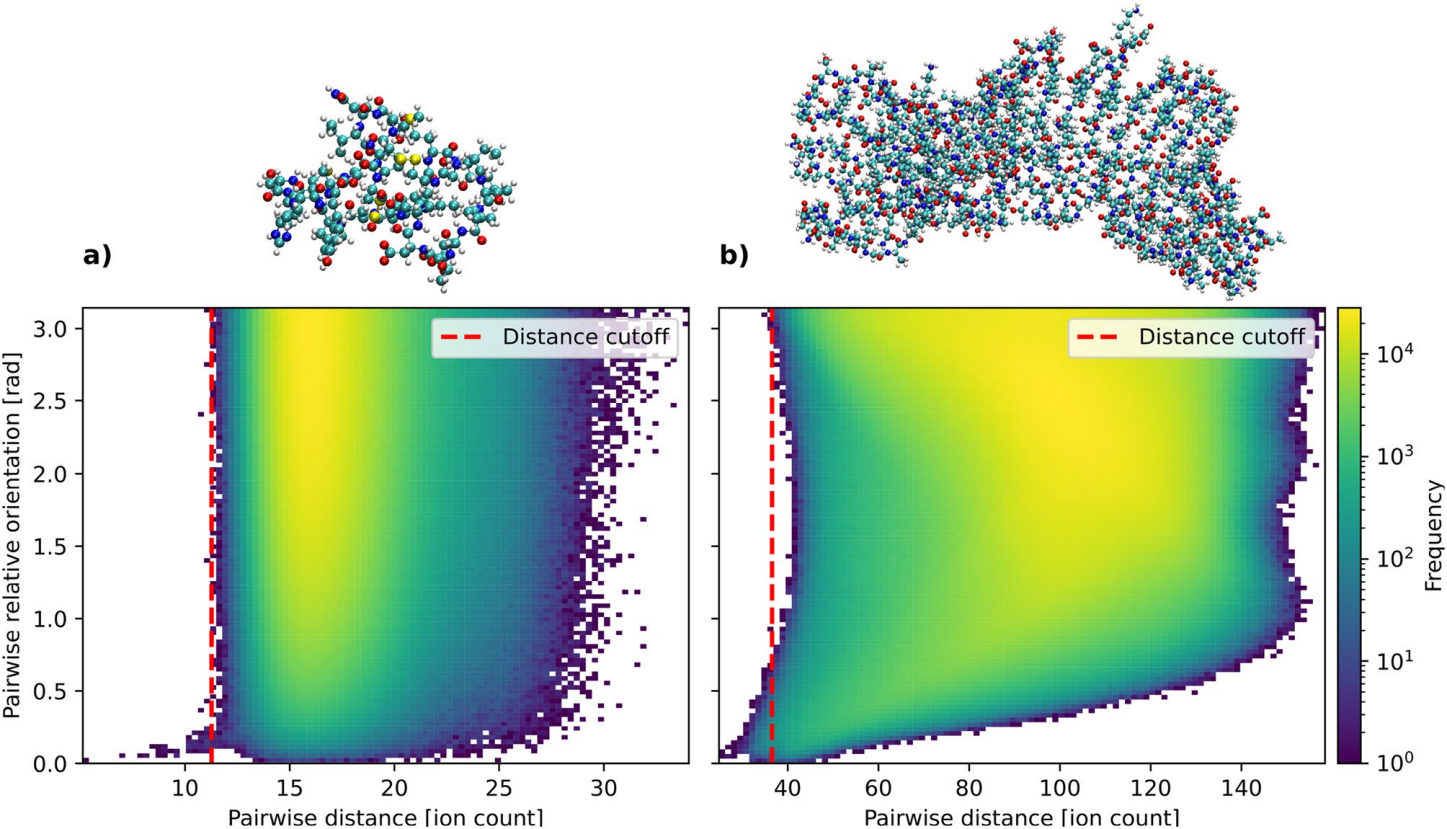
Relative orientation against distance (Eq.10) between 10000 pairs of explosion footprints from (**a**) protein with PdbID *7ely* having 375 atoms, and (**b**) protein with PdbID *2ol6* having 3640 atoms. The frequency represents the number of ion explosion pairs at a fixed pairwise distance and relative orientation. There is no global correlation but there is a trend in the regime under the distance cutoff. We can see a sharp saturation of the orientation, which we take as the distance cutoff, where pairs of ion maps below the distance cutoff have close relative orientation. Earlier studies indicate that a biased orientation with a standard deviation of 60 degrees (corresponding to 1 rad), is sufficient for the EMC algorithms to converge more reliably.^24^.

## Results

In the following section, we will show how to infer the relative orientation of pairs of proteins from our virtual detector explosion footprints. The detector footprints is a 2D spatially resolved histogram, counting how many ions fall into each bin (pixel), the detector image does not contain any information about momentum or atomic species, only the spatial distribution. Figure 1 shows a schematic of our simulated experiment, with two flat detectors that capture the ions ejected from a protein explosion using a Monte Carlo/Molecular Dynamics (MC/MD) software^25^. In the first step, using 10000 explosion footprints for two proteins (PdbID: *7ely* and *2ol6*) as an example, we can calculate the pairwise distance between footprints (as the Euclidean norm in pixel space, see Eq. 10 in Methods) among all pairs of footprints and then plot it against the ground truth pairwise relative orientation (Fig. 2), where relative orientation is given by the minimal orientation angle, $\theta$, needed to align to axes $n_A, n_B$ in 3D, given by

1 $$\begin{aligned} n_A \cdot n_B = \cos \theta . \end{aligned}$$

We see that for many patterns there is no correlation, which is expected since most orientations will not be close to each other when chosen at random. Pairs of footprints with small distance correlate with small relative orientation between the proteins. A distance cutoff enables selection of similarly oriented pattern pairs. We will next show how this distance cutoff can be predicted based on the number of atoms in the sample and without any prior knowledge of the orientation. Noting the distribution of pairs in this space, Fig. 2, we see that only a small fraction of pattern pairs exhibit close alignment, as expected from random uniform sampling over the SO(3) group, the rotation group in three-dimensional space, for all possible rotations.

To define the distance cutoff for a given protein we take the upper envelope of the bins in the orientation-distance distribution (2), resulting in the curve

2 $$\begin{aligned} f(x) = \max \{y\,|\,(x,y)\,\mathrm {occupied\,by\,at\,least\,one\,sample}\}, \end{aligned}$$

where $x$ is the pairwise distance and $y$ the pairwise relative orientation. Then the distance cutoff, $x_\textrm{cutoff}$, is given by $x$ where maximum value of the derivative of $f$ is obtained,

3 $$\begin{aligned} x_\textrm{cutoff} = \mathrm {arg\,max}\left\{ \frac{{\textrm{d}}{}}{{\textrm{d}}{x}}f(x)\right\} . \end{aligned}$$

**Fig. 3:**
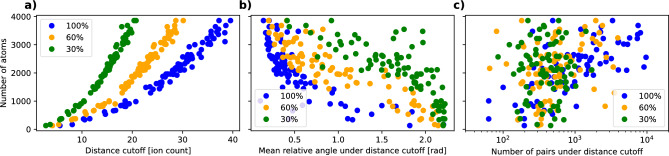
Various properties in relation to number of atoms in the sample, with different levels of detector efficiency at 30%, 60%, and 100%. (**a**) Distance cutoff required. (**b**) Mean relative angle for all the pairs under the distance cutoff. (**c**) Number of pairs under the distance cutoff. There seems to be a clear trend for larger distance cutoff, tighter angular restrain, and number of pairs under the distance cutoff with regards to the mass.

In the second step, our analysis of 85 different proteins with a size range of 100–4000 atoms, with 10000 explosion footprints per protein, reveals that the required distance cutoff is not universal but is strongly influenced by the number of atoms in the protein. Figure 3 illustrates the value of the distance cutoff, average relative orientation, and number of pairs under the distance cutoff in relation to the number of atoms in all the 85 investigated proteins.

We note that, in general, smaller proteins tend to require a smaller distance cutoff, and both the angular spread and number of matched pairs under the distance cutoff tend to be lower for smaller proteins. However, the number of atoms is not the only factor influencing the distance cutoff values. Proteins with a similar number of atoms show a slight spread in their distance cutoff points, which likely is due to differences in their structural symmetries. These symmetries make the explosion footprint look very similar from different angles, affecting the distance cutoff values. For example, the patterns from a protein with 2-fold symmetry would be indistinguishable if we rotate it by $\pi$ around the axis of symmetry. We observe that the relative orientation of protein pairs under the distance cutoff improves for larger proteins, giving tighter orientation between the pairs on average. The number of pairs under the distance cutoff also significantly increases with larger proteins, displaying an exponential increase in relation to the number of atoms in the sample.

**Fig. 4:**
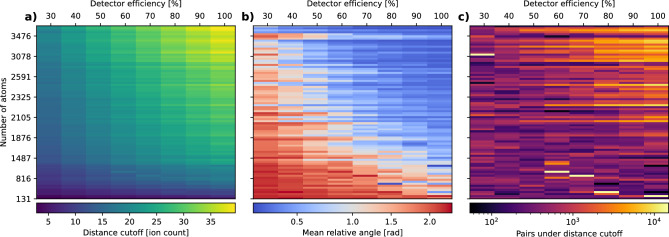
(**a**) Distance cutoff, (**b**) mean relative angle for all the pairs under the distance cutoff, and (**c**) number of pairs under the distance cutoff, in relation to the total number of atoms in the sample (on y-axis), with different levels of detector efficiency scaling from 30% to 100% (on top x-axis). The trend implies that more ions measured on the detector (regardless if from protein size or detector efficiency) give better results for (**a**–**c**). Note that with increasing number of ions, not only a tighter angular restrain is achieved, but also the number of pairs that we can match increases.

So far we have assumed that all the ions that hit the detector are registered, in a real experiment this is most likely not the case. Here we carry out the same analysis assuming that we lose some ions. To do this we define a detector efficiency as the probability that an ion hitting the detector is recorded. We calculate this for efficiencies between 30% and 100%. The results are presented in Fig. 4, the rows are the different proteins, sorted by mass, and the different columns are the different levels of the detector efficiencies. The different colorbars represent the distance cutoff value, average relative angle under the distance cutoff and the number of pairs under the distance cutoff. Note that the proteins are uniformly distributed in mass, so the mass axis is not linear.

## Discussion

In this simulation study, we employed a Monte Carlo/Molecular Dynamics (MC/MD) model to explore the use of ion explosion patterns for retrieving partial orientation information during SPI experiments at XFELs. Our simulations demonstrate that ion trajectories encode relative orientation between protein pairs, offering a novel and complementary route for orientation retrieval. Notably, this study does not attempt to use the explosion patterns for full orientation reconstruction, rather, we show that such information is present and that future algorithms could feasibly exploit it to improve orientation alignment.

**Fig. 5:**
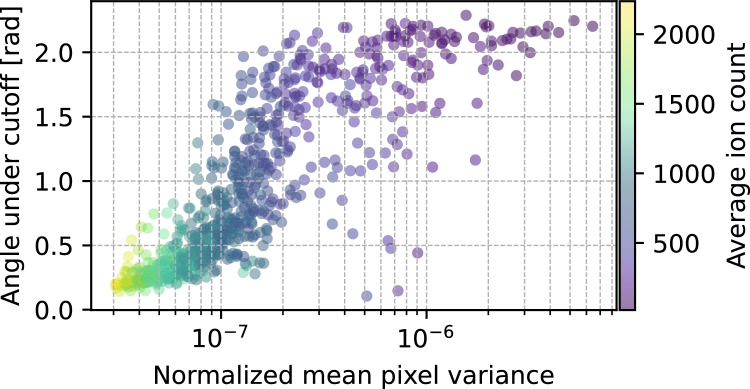
Average relative angle under threshold achieved for the rotated proteins against the variance in patterns for unrotated proteins. Each point represents a combination of one of the 85 proteins and a detector efficiency. We calculate this for efficiencies between 10% and 100% with steps of 10%, giving us a total of $10\cdot 85$ points. The color scale shows the average number of ions measured on the detector. This visualizes two important things, number of detected ions (regardless if from protein size or detector efficiency) is linked to the explosion reproducibility, and reproducibility is linked to the achieved angular restrain.

By analyzing the distance distribution between explosion footprints, we propose a method to identify protein pairs with similar orientations. We systematically investigated how the distance cutoff threshold, angular accuracy, and number of matching pairs vary with protein size and detector efficiency (Fig. 4). Our results show that performance improves with protein size. We attribute this to two main factors: (1) larger proteins produce more ions, increasing the signal, and (2) the reproducibility of explosions increases due to stronger and more deterministic Coulombic forces in high-charge-density systems. In contrast, smaller proteins exhibit higher variance since ionization pathways play a bigger role. This is confirmed in Fig. 5, which shows that average pixel variance of explosion patterns of proteins with the same orientation decreases with protein size. Consequently, for small proteins, similar orientations do not always yield similar footprints in pixel space, complicating reliable orientation matching. Note that in both Fig. 4b) and Fig. 5, the orientation error saturates at roughly 2.2 rad. This saturation is expected and can be explained analytically. The probability distribution of angular distance (Eq. 1) in SO(3) is given by^26^,

4 $$\begin{aligned} p(\theta ) = \frac{1}{2}\sin ^2(\frac{\theta }{2}). \end{aligned}$$

The median of this distribution can be found by integrating the probability density,

5 $$\begin{aligned} \int _0^{\theta _{\textrm{median}}} p(\theta ) d\theta = \frac{1}{2}, \end{aligned}$$

this gives the expression

6 $$\begin{aligned} \frac{\theta _{\textrm{median}}}{2} - \frac{\sin (2\theta _{\textrm{median}})}{4} = \frac{1}{2}, \end{aligned}$$

which can be solved numerically to get $\theta _{\textrm{median}} \approx 2.24$ rad. Thus, when the average angle reaches around 2.2 rad, it is saturated and the orientations are essentially random.

The model for the virtual detector used in this study could plausibly be implemented with a position-sensitive microchannel plate (MCP) detector. Previous experiments have used MCPs to detect explosion patterns from smaller molecules^18^. The detector in Korkulu *et al*.^27^ shares the same dimensions as ours, but with 500 $\mu$m resolution. Treating each pixel in our virtual setup as an MCP channel yields a coarser 6000 $\mu$m resolution, an order of magnitude larger, and likely easier to construct experimentally. The geometric and physical parameters studied here are thus within feasible experimental reach.

SPI has steadily progressed toward smaller biological systems. After the landmark 400 nm Mimivirus reconstruction in 2011^28,29^, SPI achieved 3D reconstructions of the PR772 bacteriophage^30^, and recently detected single-particle diffraction from the 14 nm chaperonin GroEL^5^. While these are significant milestones, pushing the size limit further remains challenging due to weak scattering signal, frequent out-of-focus hits, and background noise. These problems will become increasingly important as the samples get smaller and will affect their orientation retrieval.

**Fig. 6:**
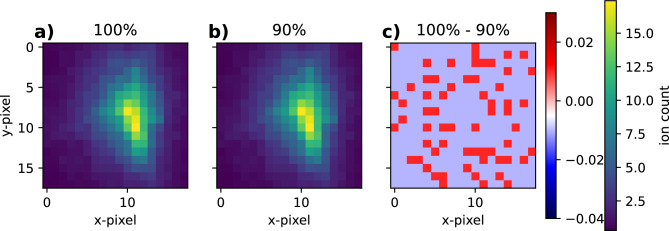
Average of 100 explosion simulations with fixed orientation of the *2ol6* protein (Fig. 2b), with (**a**) 100% incident fluence, (**b**) 90% incident fluence, and (**c**) the difference map between $100\%-90\%$. At these fluences, there is very little variance in the ions trajectories due to the change in fluence, the majority of variance arise from structural heterogeneity, which is present in both cases.

In our study we have assumed a constant XFEL photon fluence, however, in real experiments the fluence fluctuates on a shot-to-shot basis. The XFEL parameters used in our simulation result in the protein becoming completely atomized, this is usually achieved when reaching just above +1 average ionization per atom. At this level, all bonds are broken and the trajectories tend to be similar and reproducible. It is experimentally possible to filter out weak hits by measuring the scattered photons or ejected ions. However, this filtering will not be perfect and there will still be a spread in fluence. To gauge the impact of minor variations in fluence, we run 100 simulations of the *2ol6* protein with fixed orientation (Fig. 2b) at $100\%$ and $90\%$ fluence. The average explosion footprints and the errors are shown in Fig. 6. The summed absolute error is 2.1 ion counts and mean absolute error per pixel is 0.007 ion counts. At the point where the proteins are severely ionized and all the bonds are broken, the variance in the ion trajectories is dominated by the differences arising from the structural heterogeneity of the proteins rather than the change in fluence.

Incorporating orientation estimates from explosion maps could overcome some of these barriers. We show that relative orientation errors below 1.0 rad can be reliably identified using pixel space analysis (Fig. 4) for all but the smallest proteins (<800 atoms). In the case of EMC, a reconstruction is usually considered successful if the relative orientation error among diffraction measurements is below $10^\circ$ (0.17 rad). Recent studies show that *a priori* knowledge of the distribution of diffraction patterns within $60^\circ$ $\approx 1$ rad can improve EMC performance^24^. Our results suggest that it is possible to infer partial knowledge over the relative orientation of the measurements within a comparable rotation angle. For larger proteins we can reliable predict orientations within 0.1 to 0.2 rad ($6^\circ$ to $12^\circ$). In this case using ion-based estimates could enable averaging over similarly oriented patterns, boosting signal-to-noise ratio and reconstruction quality, especially for noisy or low-dose diffraction images. We hypothesize that combining ion-based orientation information with diffraction data can reduce the number of required patterns, tolerate noisier inputs, and extend SPI to smaller and more challenging targets. This would mark a significant advance in reconstruction strategies.

Leveraging ion explosion data represents a logical next step in SPI. The required ion data already exists in many experiments, and appropriate detectors are within reach. Fundamentally, SPI should be capable of Ångström-resolution reconstructions, but major challenges remain in sample delivery, algorithm development, XFEL brightness, and noise suppression^7^. We believe that incorporating ion trajectories into the orientation retrieval pipeline is a promising route toward achieving higher resolution for smaller proteins, pushing the boundaries of what is currently possible with SPI.

## Methods

### Simulation setup

All simulations are carried out using MolDStruct^22,25^, a Monte Carlo/Molecular Dynamics code implemented in GROMACS 4.5.6^31^, freely accessible (available at https://github.com/moldstruct/mc-md). The ionization model includes photoionization, fluorescence, and Auger-Meitner decay, furthermore charge transfer is included between hydrogen and other atomic species. The model assumes all photons and electrons escape and therefore does not take electron collision effects into account. This is a reasonable assumption for samples in the size range we consider^25^. The simulations are initialized and equilibrated with the CHARMM36 forcefield^32^. After energy minimization using steepest descent, we equilibrate in vacuum, at 300 K, using a 1 fs timestep, while keeping the protein’s orientation fixed. From this simulation, we sample structural variations in the protein that serve as initial configurations for the ionization simulations. In total we run 100 simulations with different initial structures for each of the 85 different proteins. The proteins are sampled from the biological assemblies deposited in the Protein Data Bank in Europe (PDBe)^33^ within the range of 100 – 4000 atoms. We limit our study to proteins in this range as they are small enough to allow the free electrons to escape, which is a requirement for our simulation code to give accurate results. We assume particle injection speed is negligible compared to the speed of the ions during the Coulomb explosion, as typical injection speeds are on orders of $10^1$-$10^2$ m/s while the ions in our simulations are ejected at $10^{4}$-$10^{5}$ m/s. The Coulomb explosion simulations are carried out with a time step of 1 as. The simulated XFEL pulse is Gaussian-shaped with a peak at $t=20$ fs and a FWHM of 10 fs. The pulse has a fluence of $5\cdot 10^{6}$ photons/nm$^2$ and photon energy 2 keV. These parameters are experimentally feasible at the SQS endstation at the European XFEL^34^. The simulations run for 250 fs, after which the total energy in the systems consists of kinetic energy, indicating that there is little to no more repulsion between most of the ions.

### Virtual detector

By the end of the explosion simulations, ion interactions have effectively ceased as they are now separated by large distances and continue to expand into space. From this point onward, their trajectories should follow relatively straight paths, allowing us to trace the direction of each ion to determine which pixel on the detector it will hit. We model the detector efficiency, defined as the probability that an ion hitting the detector is registered, using a factor between 0.0 and 1.0. To capture the ions ejected from the Coulomb explosion, we envision placing two flat, square detectors, each with a side length of 120 mm, positioned 30 mm from the origin of the explosion on opposite sides, shown in Fig. 1. We uniformly distribute $18\times 18$ points on this surface, functioning as pixels for the detector.

By combining the footprints from both detectors, we create a new image represented as a $(2\cdot 18\times 18)$ matrix. We extract the displacement vector of each ion and follow each ions path, each ion that reaches the detector adds one count to the nearest bin, ions outside the detector range are discarded. The resulting pattern, the explosion footprint, can be viewed as a 2D image, as seen on the face of the detectors in Fig. 1.

When calculating the variance of the image tensor $I^p_{k,i}$ (for protein $p$, image $k$, and pixel $i$), we first normalize each image by its total intensity

7 $$\begin{aligned} \hat{I}^p_{k,i} = \frac{I^p_{k,i}}{\sum _{i=1}^n I^p_{k,i}}, \end{aligned}$$

the variance for each pixel in pattern $k$ for protein $p$ is then given by

8 $$\begin{aligned} \sigma _{p,i}^2 = \operatorname {Var}(\hat{I}^p_i) = \frac{1}{K} \sum _{k=1}^K \left( \hat{I}^p_{k,i} - \mu ^p_i \right) ^2, \quad \text {with} \quad \mu ^p_i = \frac{1}{K} \sum _{k=1}^K \hat{I}^p_{k,i}, \end{aligned}$$

where $K$ is the total number of footprints. Finally we take an average over all variances for each pixel $i$ to get the variance per protein

9 $$\begin{aligned} \sigma _p^2 = \frac{1}{N}\sum _{i=1}^N\sigma ^2_{p,i}, \end{aligned}$$

where $N$ is the total number of pixels. Note that we are calculating the variance individually for all different proteins, we are not computing inter-protein variances.

### Orientation retrieval

All simulations are carried out with the same orientation, in the analysis we will instead consider relative orientation between pairs of proteins. To do this we sample *N* 3D-rotations uniformly as unit quaternions, which is common when numerically working with rotations, and apply them to our ion vectors. This has the effect of emulating simulations with different orientations. We augment our data by extracting multiple explosion patterns from each explosion simulation, at different orientations, As an example, if we extract 10000 patterns in total from the $100$ simulations, we take $10000/100 = 100$ patterns from each simulation, each pattern at a different orientation of the protein. We use the relative orientation between two proteins to quantify how similar their orientations are. For a 3D object it is possible to decompose any set of rotations as a single rotation by one angle around some axis, thus for any pair of orientations, we can describe the difference in orientation as a single axis-angle rotation, rather than decomposing it into XYZ components. For two proteins with orientations described by the quaternions $R_i$ and $R_j$, we can extract the relative orientation between the proteins from the unit quaternions as $\text {Re}[R_iR_j^{-1}] = a$, $\theta = 2\cos ^{-1}(a)$. Repeating this for the *N* patterns extracted, we can create $N(N-1)/2$ pairs of patterns, and calculate relative orientation between each pair of proteins.

To calculate difference between pairs of detector patterns, we use the Euclidean norm in pixel space to calculate the distance between 2D ion detector hit patterns (not normalized) $(A,B)\in \mathbb {R}^{(2\cdot 18\times 18)}$, given by

10 $$\begin{aligned} d(A,B)= ||A-B||_2 = \sqrt{\sum _{i=1}^{N}(A_i-B_i)^2}, \end{aligned}$$

where $N$ is the number of pixels in the pattern. Another technique we will use is to blur our patterns by applying a convolution with a 2D Gaussian kernel, $G = \frac{1}{2\pi \sigma ^2}\exp \left( -\frac{x^2+y^2}{2\sigma ^2}\right)$ with $\sigma =0.5$ pixels. This will slightly smear out the patterns which is useful for allowing some wiggle room when measuring similarity of explosion patterns. Visually similar but slightly misaligned patterns may otherwise rank as dissimilar in pixel space. This smoothing improves robustness against small spatial offsets.

## Data Availability

Data is provided within the manuscript.
